# Early detection and monitoring of cerebral ischemia using calcium-responsive MRI probes

**DOI:** 10.1073/pnas.1908503116

**Published:** 2019-09-23

**Authors:** Tanja Savić, Giuseppe Gambino, Vahid S. Bokharaie, Hamid R. Noori, Nikos K. Logothetis, Goran Angelovski

**Affiliations:** ^a^MR Neuroimaging Agents Group, Max Planck Institute for Biological Cybernetics, 72076 Tuebingen, Germany;; ^b^Neuronal Convergence Group, Max Planck Institute for Biological Cybernetics, 72076 Tuebingen, Germany;; ^c^Department of Physiology of Cognitive Processes, Max Planck Institute for Biological Cybernetics, 72076 Tuebingen, Germany;; ^d^Department of Imaging Science and Biomedical Engineering, University of Manchester, Manchester M13 9PL, United Kingdom

**Keywords:** calcium, contrast agents, ischemia, magnetic resonance imaging

## Abstract

Cerebral ischemia is one of the leading causes of mortality and disability in infants and adults and its timely diagnosis is essential for an efficient treatment. We present a methodology for fast detection and real-time monitoring of fluctuations of calcium ions associated with focal ischemia using a molecular functional MRI approach. We used a dinuclear paramagnetic gadolinium(III) complex chelate that changes MR image contrast through its reversible interaction with extracellular calcium ions, while applying a remote transient middle cerebral artery occlusion as a model for ischemic stroke. Our method sensitively recognizes the onset and follows the dynamics of the ischemic core and penumbra with submillimeter spatial and second-scale temporal resolution, thus paving the way for noninvasive monitoring and development of targeted treatment strategies for cerebral ischemia.

## Significance

The duration of cerebral ischemia is a key factor in determining the severity of brain damage and the course of action. Thus, an accurate and timely observation of the ischemic process is highly critical. Here we present a molecular neuroimaging approach that enables direct detection and real-time visualization of transient cerebral ischemia. The method relies on high-resolution observation of extracellular calcium alterations associated with the spatiotemporal dynamics of cerebral ischemia, using a selective molecular MRI probe. The rapid detection of calcium fluctuations in healthy and disease states will not only lead to essential insights for successful treatment and recovery of ischemic brain tissue but will also improve our understanding of the underlying neurobiology of neurological and psychiatric disorders.

Cerebrovascular diseases rank as the second-leading cause of mortality, accounting for 9.6% of all deaths worldwide (1, 2). For clinicians, the most relevant aspect is likely the identification of portions of the ischemic tissue that are still potentially reversible (3). Understanding the complex pathophysiology of focal cerebral ischemia requires the use of reproducible experimental models to characterize the ischemic penumbra (4). Thus, accurate and timely detection, visualization, and monitoring of the spatiotemporal course of ischemia are of great practical relevance in the treatment and recovery of ischemic injuries (5, 6).

The most commonly used imaging techniques for ischemia diagnosis in infants and adults are ultrasonography and computed tomography. However, they often lack the necessary sensitivity to detect ischemia at an early stage (7). To this end, ^18^F-based positron emission tomography imaging probes of mitochondrial complex I activity were developed as specific markers of the neuronal death caused by ischemia (8, 9). Still, magnetic resonance imaging is considered a more reliable neuroimaging technique, as it allows a better differentiation of the damaged regions at earlier stages of ischemic injury (10); it also does not require administration of radioactive tracers. Nonetheless, the current MRI-based technologies, characterized by great spectral sensitivity, spatial localization, and potentially quantitative tracking of changes in the concentration of endogenous substances, are only partially exploited in functional studies. Understanding of dynamic, site-specific, and temporally differentiated processes, such as that of tissue ischemia, may still progress significantly with greater use of MRI.

Cerebral ischemia, specifically, results in a reduced blood supply to the brain tissue, causing oxygen-glucose deprivation and adenosine triphosphate (ATP) production failure. The resulting energy crisis can trigger a cascade of detrimental biochemical and physiological events, including strong disturbances in calcium homeostasis, leading to acute or delayed cell death (11). These physiological changes can then be detected by means of a single MRI technique, such as diffusion- and *T*_2_-weighted MRI or ^23^Na MRI, however only after at least 10 min or hours after the ischemic onset (12, 13). By combining a pair of MRI-based methods in specific perfusion- and diffusion-weighted imaging (PWI and DWI, respectively) into a so-called perfusion–diffusion mismatch, an ischemic penumbra (IP) could be differentiated from the ischemic core. Despite these advances, recent studies have shown that this model is only an approximation of the IP (14). Concurrently, functional MRI (fMRI) techniques based on the blood oxygenation level-dependent (BOLD) signal have also been used for the identification of the core infarct and penumbra regions in subjects affected by acute ischemic stroke (15), or in the assessment of impairment of the executive functions and frontoparietal network connectivity as later consequences of the ischemic event (16).

Nonetheless, it has been shown that, upon ischemia, the extracellular calcium concentration ([Ca^2+^]_e_) decreases dramatically and, in the case reperfusion could be promptly established, [Ca^2+^]_e_ returns to its resting value (17, 18). Changes in cerebral calcium concentration can therefore provide a marker for monitoring the intensity and duration of ischemic injuries. Traditionally, measurements of [Ca^2+^]_e_ have only been performed locally using calcium-sensitive microprobes (19). In recent years there has been substantial progress toward the development of molecular markers capable of monitoring changes in calcium concentration using fMRI. Yet, such early studies have been focusing mostly on technological optimization and advancements, while the most recent reports involved development of a few very potent functional probes of different chemical origin and size (20–23).

**Fig. 1.**
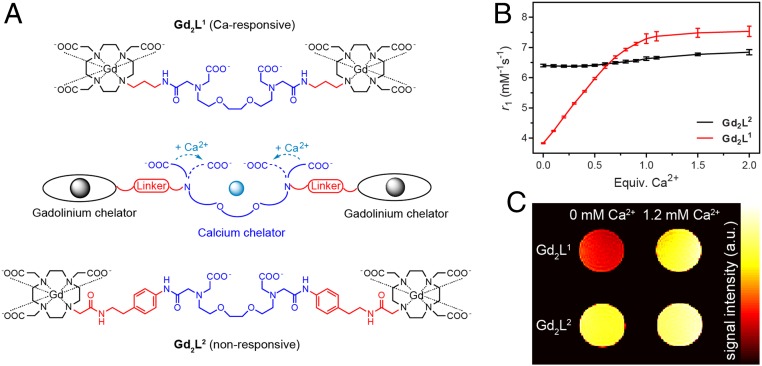
Detection of Ca^2+^ concentration changes with responsive MRI probes. (*A*) Molecular structures of the responsive **Gd**_**2**_**L**^**1**^ (*Top*) and the control, nonresponsive **Gd**_**2**_**L**^**2**^ (*Bottom*) with the interaction mechanism of the SCA with Ca^2+^ (*Middle*). (*B*) Proton relaxation enhancement titration of **Gd**_**2**_**L**^**1**^ and **Gd**_**2**_**L**^**2**^ (1.0 mM Gd^3+^) measured at 37 °C and 7T in buffered medium (Hepes, 50 mM, pH 7.4). (*C*) In vitro MRI on tube phantoms: **Gd**_**2**_**L**^**1**^ and **Gd**_**2**_**L**^**2**^ (2.5 mM Gd^3+^) without and with 1 equivalent of Ca^2+^ (1.2 mM) in Hepes (50 mM, pH 7.4).

To further expand the scope of this approach and demonstrate its favorable applications in neuroimaging, we utilized the calcium-sensitive MRI contrast agent as the molecular fMRI biomarker. This molecular probe is a bismacrocyclic gadolinium(III) complex, **Gd**_**2**_**L**^**1**^, that bears a common EGTA-derived calcium chelator (EGTA, ethylene glycol tetraacetic acid) acting as a so-called smart contrast agent (SCA). Such an agent is able to selectively interact with Ca^2+^ ions, discriminating other relevant endogenous bivalent ions, as it has been determined with competitive titrations and experiments in cellular model systems (24, 25). Moreover, this dinuclear paramagnetic chelate is specifically designed to reversibly interact with calcium ions, which trigger intramolecular conformational changes and affect the longitudinal *T*_1_ relaxation time of surrounding water, thereby changing the longitudinal relaxivity (*r*_1_) of tissues and generating a [Ca^2+^]-dependent MR image contrast (Fig. 1). In addition to the responsive probe, we developed an analogous nonresponsive chelate, **Gd**_**2**_**L**^**2**^ (Fig. 1*A* and *SI Appendix*, Fig. S1), to serve as a control and comparison with the performance of **Gd**_**2**_**L**^**1**^ in terms of the efficacy and selectivity. More specifically, we designed a molecule that bears the same DO3A-type chelator for Gd^3+^ (DO3A, 1,4,7,10-tetraazacyclododecane-1,4,7-tricarboxylic acid) and the same EGTA-type chelator for Ca^2+^. However, by selecting different linkers, we intentionally interrupted any communication between these two chelating moieties in **Gd**_**2**_**L**^**2**^, thereby expecting no changes in the *T*_1_ along with changes in calcium concentration.

Here, we used these molecular probes to execute a series of fMRI experiments while inducing the ischemic stroke in vivo. Such an event triggered fluctuations of calcium concentration associated with cerebral ischemia and enabled its detection and monitoring with excellent spatiotemporal resolution.

## Results

The in vitro experiments in buffered media showed a significant increase of *r*_1_ for **Gd**_**2**_**L**^**1**^ upon the addition of calcium ions and virtually no change for **Gd**_**2**_**L**^**2**^ under the same conditions (Fig. 1*B*). More precisely, ^1^H NMR relaxometric titrations were performed at 7T and 37 °C, and the variations occurring in *T*_1_ after the addition of Ca^2+^ ions to a solution containing **Gd**_**2**_**L**^**1**^ or **Gd**_**2**_**L**^**2**^ were measured. The results obtained displayed an overall increase of 100% in *r*_1_ in the case of **Gd**_**2**_**L**^**1**^, which reduces to still a >50% increase in *r*_1_ using the cell-culture medium (25). As a consequence, such changes in *r*_1_ were expected to reflect on generating lower *T*_1_-weighted MRI signal at low calcium concentrations and higher signal as the concentration increases. On the other hand, no significant variation in *r*_1_ was observed in the case of **Gd**_**2**_**L**^**2**^; therefore, we expected *T*_1_-weighted MRI signal produced by **Gd**_**2**_**L**^**2**^ to remain insensitive to any calcium concentration changes. To confirm this observation, we performed the MRI tests on tube phantoms at ambient temperature in the scanner at the same magnetic field (7T). The *T*_1_-weighted images acquired for a set of samples containing either **Gd**_**2**_**L**^**1**^ or **Gd**_**2**_**L**^**2**^ in the presence or absence of Ca^2+^ at physiological concentration confirmed the behavior observed with the ^1^H NMR relaxometric titrations. Namely, the tubes containing **Gd**_**2**_**L**^**1**^ produced very different MRI signal responses, where significantly higher *T*_1_-weighted signal was obtained for the sample containing Ca^2+^. Concurrently, the MRI signal intensities produced by the tubes containing **Gd**_**2**_**L**^**2**^ were identical, and therefore not influenced by the presence or absence of Ca^2+^ (Fig. 1*C*).

**Fig. 2.**
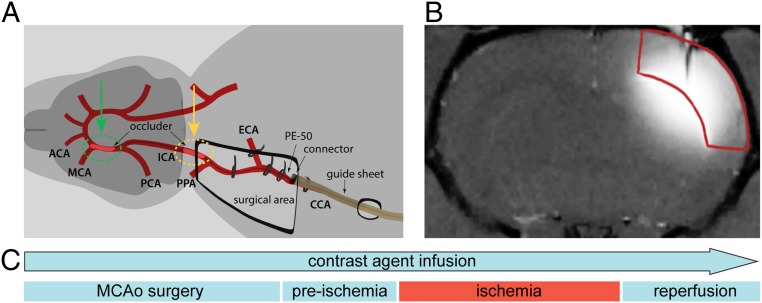
Preparation of tMCAo for molecular fMRI studies. (*A*) Surgical area and interventions on the rat for tMCAo. The preparation for the remotely induced and controlled tMCAo was carried out introducing a silicone-coated occluder through support tubing connected to the intraarterial catheter fixated inside the common carotid artery. The occluder was advanced in the direction of the right internal carotid artery, until 2 mm after bifurcation with the pterygopalatine artery (pre- and postischemia period; marked with a yellow dashed circle; *SI Appendix*, Fig. S2, *Left*); subsequent positioning of the occluder during the ischemia induction is marked with a green dashed circle (*SI Appendix*, Fig. S2, *Right*). (*B*) Infusion of the SCA (seen as the hyperintense region) in the rat somatosensory cortex (marked in red). At first, this methodology involved performing the continuous intracranial infusion of **Gd**_**2**_**L**^**1**^ or **Gd**_**2**_**L**^**2**^ in the somatosensory cortex of adult rats, using an s.c. positioned osmotic pump, before any tMCAo-related phase. (*C*) Scheme representing the experimental procedure until the end of the reperfusion period. Once the animal was positioned inside the 7T MRI scanner, an imaging protocol consisting of a series of *T*_1_-weighted MR acquisitions with a duration of 19.9 s was executed every 2 min. Each experiment was divided into 3 segments: preischemia, ischemia, and reperfusion period. Transient ischemia was induced by advancing the occluder for 6 to 8 mm until resistance was felt, meaning that the occluder reached the anterior carotid artery and thus occluded the MCA, while reperfusion was allowed using the reverse action.

To demonstrate the appropriateness of our molecular fMRI technique for monitoring pathophysiological processes such as transient cerebral ischemia, we chose a remote transient middle cerebral artery occlusion (tMCAo) experimental protocol (Fig. 2) (26).

The MCAo is a representative model to study molecular mechanisms of brain injury, since ∼70% of human ischemic strokes are caused by an occlusion of the MCA and its branches (27, 28). By choosing this particular model for demonstrating the appropriateness of our approach in vivo, we secured 2 highly valuable advantages for the visualization of a variation in [Ca^2+^]_e_ triggered by physiological alterations. The first advantage resides in the adoption of a transient occlusion of the MCA. By doing so, we were able to trigger the ischemia at will and monitor the resulting drop in [Ca^2+^]_e_; thereafter, the reperfusion of the tissue consequently recovered the preocclusion [Ca^2+^]_e_. In this fashion the timing of the generated physiological processes is fully controlled, with clear onset and offset points that are in correspondence with the perturbations of the *T*_1_-weighted MRI signal. The second advantage derives from performing the MCAo procedure remotely. This particular feature results in the possibility of inducing the MCAo in the MRI scanner directly. Subsequently, this approach enabled immediate monitoring of changes in the calcium concentration upon ischemia and reperfusion, by recording *T*_1_-weighted MRI without any delays or temporal discontinuity; moreover, it allowed us to maintain the position and orientation of the voxels in the region of interest (ROI), which greatly simplified the data analysis phase.

The above-mentioned setup required a continuous infusion of SCA, achieved by implanting s.c. an Alzet osmotic pump filled with **Gd**_**2**_**L**^**1**^ and **Gd**_**2**_**L**^**2**^. With such an administration method initiated before tMCAo surgery preparation, we were able to begin with functional MRI acquisitions immediately after the animal was transferred inside the MRI scanner. Moreover, selecting this method instead of a single direct intracranial injection allowed us to follow the effect of these SCAs for longer periods as required by the experimental setup, without concerns of observing a decay in the MRI signal due to SCA washout. Namely, the continuous delivery of the CAs throughout the experiment compensates for the fast washout of the MRI probes in the tissue, thus overcoming one of the critical obstacles to the in vivo application of small- and moderate-sized SCAs (<∼2 kDa) (29).

The resulting spreading of the SCA and subsequent analysis of the ROIs resulted in large volumes where ischemia caused by MCAo can be observed. This is much larger than possible to achieve by means of conventional electrophysiology with calcium-sensitive electrodes (19, 30).

Based on the benefits at hand, the procedure was applied for both contrast agents **Gd**_**2**_**L**^**1**^ and **Gd**_**2**_**L**^**2**^ (*n* = 5 animals per agent). In addition, control experiments with **Gd**_**2**_**L**^**1**^ and **Gd**_**2**_**L**^**2**^ (*n* = 5 animals per agent) without the transient ischemia induction were conducted in order to demonstrate that, if the occlusion is not performed, no *T*_1_-weighted MRI signal perturbation occurs other than that related to the infusion of the CA.

**Fig. 3.**
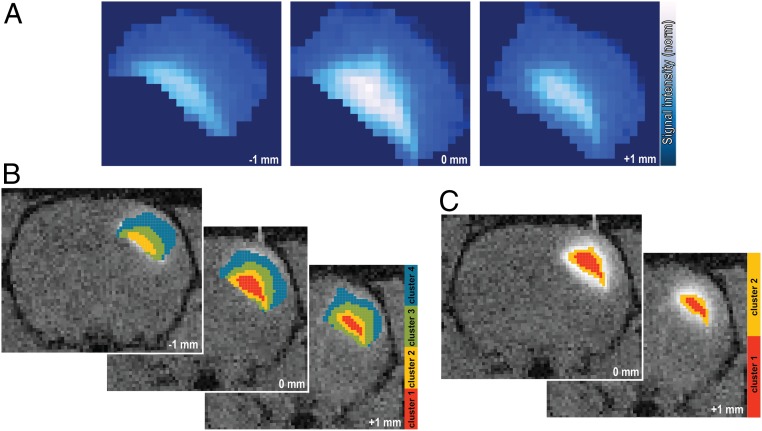
Data analysis upon the SCA infusion. (*A*) Coverage of the SCA enhanced *T*_1_-weighted MRI signal. (*B*) *K*-means cluster maps of the *T*_1_-weighted signal using responsive **Gd**_**2**_**L**^**1**^ infused in the rat somatosensory cortex. (*C*) Hierarchical cluster maps of the *T*_1_-weighted signal using responsive **Gd**_**2**_**L**^**1**^.

Raw *T*_1_-weighted MR images showed that, with this approach, an ROI of more than 3 mm could be covered (Fig. 3*A*). *K*-means clustering on masked normalized *T*_1_-weighted images suggested concentric patterns of SCA propagation in the brain tissue (Fig. 3*B*). Concurrently, centroids of clusters closer to the epicenter of injection result in higher values on average, which is a reflection of the higher local probe concentration. This qualitative behavior can be seen in all experiments and was not affected by variations in the choice of number of clusters (*SI Appendix*, Figs. S4 and S5). We used a hierarchical clustering scheme which led to ROIs of comparable sizes, even when we started with initial masks containing wildly different numbers of voxels. The above-mentioned qualitative behavior is maintained in the resulting ROIs (Fig. 3*C* and *SI Appendix*, Fig. S6).

**Fig. 4.**
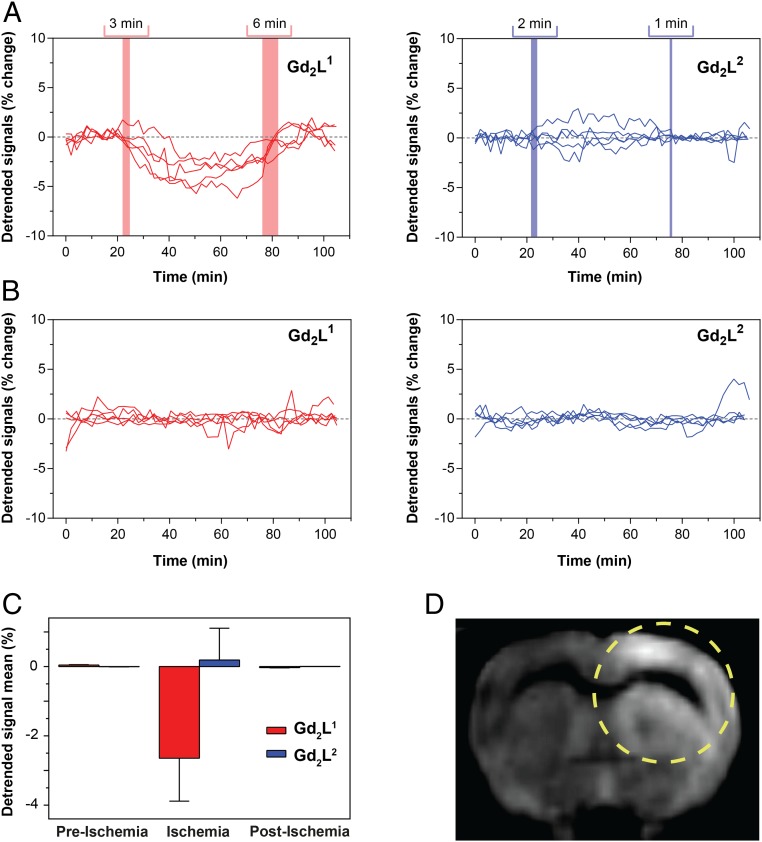
Molecular fMRI of ischemia with Ca-sensitive MRI probes. (*A* and *B*) Detrended signals of the experiments with **Gd**_**2**_**L**^**1**^ (*Left*) or **Gd**_**2**_**L**^**2**^ (*Right*) with tMCAo induction (*A*) and the control experiments with **Gd**_**2**_**L**^**1**^ (*Left*) or **Gd**_**2**_**L**^**2**^ (*Right*) without tMCAo induction (*B*). The colored regions in *A* indicate time periods when the MCAo was initiated (*Left*) or finished (*Right*) (time range combines all independent experiments). (*C*) Average values of detrended signals during the recordings with **Gd**_**2**_**L**^**1**^ and **Gd**_**2**_**L**^**2**^ with tMCAo induction in the 3 segments (preischemia, ischemia, and postischemia). (*D*) Ischemia confirmation by means of DWI acquired for the MCAo experiment; a yellow dashed circle marks the area affected by stroke.

Using an algorithm which comprises a customized detrending scheme (*SI Appendix*, *Methods* and Fig. S7), we aimed to highlight the differences in responses for the 2 different molecules during the remote tMCAo experiments (Fig. 4 *A* and *B*).

The results show a clear distinction between signals obtained from **Gd**_**2**_**L**^**1**^ and from **Gd**_**2**_**L**^**2**^ when tMCAo is induced. In agreement with previous studies documenting a reduction of [Ca^2+^]_e_ during cerebral ischemia, the intensity of the *T*_1_-weighted MRI signal promptly decreased due to the interaction of **Gd**_**2**_**L**^**1**^ with extracellular calcium, reaching up to 5% of difference in detrended signal. Moreover, the signal was recovered as the brain tissue was reperfused (Fig. 4*A*, *Left*). The responsiveness of **Gd**_**2**_**L**^**1**^ for calcium fluctuations in vivo was further demonstrated by the behavior of the *T*_1_-weighted MRI signal in the presence of the control probe **Gd**_**2**_**L**^**2**^. The signal intensity remained unperturbed by the onset and recovery of ischemia (Fig. 4*A*, *Right*). Likewise, control experiments performed with both CAs without induction of tMCAo showed similar results as for **Gd**_**2**_**L**^**2**^ under tMCAo conditions (Fig. 4*B*). The mean values of the detrended signals in the ischemia experiments under different conditions demonstrated the appropriateness of our method to detect the onset and time course of ischemic injury (Fig. 4*C* and *SI Appendix*, Tables S1 and S2). Namely, while using different calcium-chelating molecules in the presence or absence of a triggered stimulation, the changes in *T*_1_-weighted MRI signal occurred only during the tMCAo experiments and only when the responsive probe was infused, at the time segments that correspond to ischemia induction and reperfusion. The control probe **Gd**_**2**_**L**^**2**^ did not exhibit the same behavior and trends. The successful induction of ischemia was confirmed at the end of the experimental procedure by applying standard diffusion- and *T*_2_-weighted imaging protocols (Fig. 4*D* and *SI Appendix*, Fig. S3) (31, 32).

## Discussion

We employed 2 paramagnetic probes, **Gd**_**2**_**L**^**1**^ and **Gd**_**2**_**L**^**2**^, which were anticipated to interact with Ca^2+^. While only **Gd**_**2**_**L**^**1**^ was designed to produce MRI signal changes, **Gd**_**2**_**L**^**2**^ was developed to maintain the same size, charge, and capability to coordinate Ca^2+^ ions as **Gd**_**2**_**L**^**1**^. Thereby, it lacked the [Ca^2+^]-dependent variability of *r*_1_, making it an ideal control CA. In addition, **Gd**_**2**_**L**^**1**^ consists of 2 gadolinium ions along with a single calcium-chelating unit, thus producing double the *T*_1_ effect from 2 gadolinium ions per unit of calcium. Consequently, the resulting *r*_1_ response of **Gd**_**2**_**L**^**1**^ in the presence of Ca^2+^ was confirmed to be strong, as observed in the relaxometric as well as in the MRI experiments on tube phantoms. These results paved the way for developing a reliable molecular fMRI method that allows the monitoring of critical pathophysiological processes in the brain, especially during cerebral ischemia. However, it should be noted that MRI probes **Gd**_**2**_**L**^**1**^ and **Gd**_**2**_**L**^**2**^ are charged in both the Ca-free and Ca-bound forms; hence, the intracranial administration ensured their delivery to the target region in sufficient quantity. In perspective, the noninvasive delivery of these probes through the blood–brain barrier (BBB) could be envisioned. To this end, a few approaches including transient BBB disruption, involvement of molecules that promote receptor-mediated transport, or development of BBB-permeable probes have been evolved, providing an affirmatory basis for possible translation of such molecular fMRI probes and their use in clinical practice (20).

Following the SCA infusion and tMCAo execution, the used Ca-responsive and nonresponsive probes (**Gd**_**2**_**L**^**1**^ and **Gd**_**2**_**L**^**2**^, respectively) exhibited the anticipated and desired behaviors: The detrended MRI signals followed the dynamics of the ischemia onset and tissue reperfusion only in the case of **Gd**_**2**_**L**^**1**^ and tMCAo stimulation. Otherwise, neither **Gd**_**2**_**L**^**2**^ under tMCAo execution nor both **Gd**_**2**_**L**^**1**^ and **Gd**_**2**_**L**^**2**^ in the absence of tMCAo stimulation showed significant changes in MRI signal. The aforementioned results indicate that the use of SCAs, specifically **Gd**_**2**_**L**^**1**^, revealed additional indispensable insights into molecular processes during ischemia with outstanding temporal dynamics. The prompt decrease of [Ca^2+^]_e_ after the tMCAo onset from roughly 1.2 to 0.1 mM (18) was successfully monitored with our method, using short acquisition times (19.9 s) and high spatial resolution (250 × 250 μm^2^), which are comparable to the previous molecular fMRI studies of similar kind (21). In turn, this method enabled an instantaneous and quantitative estimation of the large area of the brain that was affected by the remote tMCAo procedure. This high (second-scale) temporal resolution and the accuracy of this imaging protocol allow for the detection of ischemia in its early stages in a dynamic manner, which is a highly critical aspect for the timely intervention and treatment of cerebral ischemia in patients. Indeed, the success of recovery of the affected brain tissue strongly depends on urgent stroke care (5). Our method rapidly provides quantitative estimates of the pace of neural circuitry loss in human ischemic stroke that enhances its potential for clinical use. In contrast, the existing MRI techniques only allow the visualization of the affected area significantly later (tens of minutes or hours after the initiation of ischemia; see the introduction). In addition, our fMRI method with a bioresponsive marker robustly visualizes dynamic changes in the tissues, particularly as demonstrated by the reperfusion of the tissue through the removal of the occlusion on the MCA. Therefore, it presents an appropriate technology to detect the exact timing of reperfusion and avoids the occurrence of false negative results as observed with comparably fast techniques such as DWI (33). It might also be envisioned that other robust dynamic alterations during neuronal activity, such as during a train of electrical stimuli that cause a [Ca^2+^]_e_ drop from 1.2 to 0.8 mM (19), could also be visualized with this methodology.

In summary, we introduced a robust tool to monitor [Ca^2+^]_e_ alterations in vivo that can enable the early detection and real-time monitoring of brain ischemic injuries. Considering that physiological changes in the damaged tissue occur rather fast, our method is ideal for the prompt detection of the ischemic onset and for revealing immediate changes during reperfusion that are crucial for the choice of therapy and subsequent recovery. The reported methodology represents a valuable addition to the current MRI methods used for the detection of cerebral ischemia. Evidently its potential applications are not limited only to the detection of ischemia, as changes in [Ca^2+^]_e_ are associated with a large number of biological processes, such as the transmission of the synaptic action potential. This molecular imaging technique could thus prove to be an essential supplement to conventional fMRI methods for the study of brain function and dysfunction, enabling direct visualization and mapping of neural activity.

## Methods and Materials

Detailed methods of the synthesis of the probes **Gd**_**2**_**L**^**1**^ and **Gd**_**2**_**L**^**2**^, relaxometric NMR titrations, and data analysis are available in *SI Appendix*.

### Magnetic Resonance Imaging.

MRI measurements were performed on a 7T Bruker BioSpec 70/30 USR magnet (software version ParaVision 5.1), using a Bruker volume coil (RF RES 300 1H 075/040 LIN/LIN TR).

#### MRI on tube phantoms with **Gd**_**2**_**L**^**1**^ and **Gd**_**2**_**L**^**2**^.

The phantom consisted of 4 capillaries (3-mm diameter, 150-µL volume) containing **Gd**_**2**_**L**^**1**^ and **Gd**_**2**_**L**^**2**^ (1.25 mM SCA = 2.5 mM Gd^3+^) with and without [Ca^2+^] (1.25 mM) in Hepes (50 mM, pH 7.4). MR images were acquired using *T*_1_-weighted imaging (fast low angle single shot [FLASH] pulse sequence) with the following parameters: repetition time (TR), 60 ms; echo time (TE), 2.95 ms; flip angle (FA), 90°; slice thickness, 2 mm; field of view (FOV), 55 × 55 mm^2^; matrix size (MTX), 256 × 256; number of excitations (NEX), 50; and total acquisition time (TA), 12 min, 48 s.

#### MRI with **Gd**_**2**_**L**^**1**^ and **Gd**_**2**_**L**^**2**^ in vivo.

MR images were acquired using *T*_1_-weighted (FLASH pulse sequence), diffusion-weighted (spin-echo echo-planar pulse sequence), and *T*_2_-weighted imaging (rapid acquisition with refocused echoes [RARE] pulse sequence). After the localizer scan was performed and the region of the **Gd**_**2**_**L**^**1**^ or **Gd**_**2**_**L**^**2**^ injection was located, the FLASH pulse sequence was optimized to cover the region of injection. The imaging parameters were: TR/TE = 80.95/1.56 ms; FA, 90°; 3 axial slices with 1-mm thickness each; FOV, 18 × 20.5 mm^2^; MTX, 72 × 82; NEX, 3; and TA, 19 s, 914 ms. The first section of MRI was acquired after the initiation of the continuous infusion of the CA (i.e., implantation of the osmotic pump; *Administration of the MRI contrast agents*) and was divided into 3 parts: preischemia (∼21 min), ischemia (∼53 min), and reperfusion period (∼31 min) by acquiring *T*_1_-weighted imaging every 2 min. Following that, DWI (TR/TE = 2,250/43 ms; slice thickness, 2 mm; interslice thickness, 2.5 mm; FOV, 32 × 29.4 mm^2^; MTX, 128 × 84; δt, 4 ms; ∆t, 17 ms; 3 diffusion directions; b values, 0, 300, 500, 1,150, and 1,400; NEX, 10; and TA, 19 min, 30 s) was acquired to confirm the ischemia. Thereafter, the infusion cannula was cut and the diffusion of the agent was monitored for 81 min by recording a series of *T*_1_-weighted images as described above. Finally, *T*_2_-weighted images were recorded using: TR/TE = 2386.1/38 ms; slice thickness, 1 mm; FOV, 24 × 24 mm^2^; MTX, 96 × 96; NEX, 40; and TA, 19 min, 5 s.

#### Animals.

The experiments were conducted on male Wistar rats (300 to 340 g; Charles River Laboratories). The animals were housed and maintained in controlled environmental conditions with a 12:12 h light–dark cycle for at least 7 d prior to the experiment, with food and water provided ad libitum. In each experiment, the animal was anesthetized with 2.5% isoflurane in O_2_ (Forene; Abbott) and then kept on 1.5 to 2.0% for maintenance. The body temperature of the animal was maintained at 37.0 ± 0.5 °C with a feedback-controlled heat pad (50-7221-F; Harvard Apparatus) and was continuously monitored by a rectal probe. All experiments with animals were approved by the local authorities (Regierungspräsidium Tübingen).

#### Administration of the MRI contrast agents.

The animal was placed in the stereotaxic frame (Stoelting). Craniotomy was performed using a manual drill (medial-lateral, 4.7; anterior-posterior, −0.5) and the dura was removed. Anchoring for the infusion cannula of the continuous pump was made with the 2-component dental adhesive (OptiBond FL; Kerr) and dental cement (Charisma Flow A1; Heraeus Kulzer) covering the surface of the skull, except the site of the craniotomy. Thereafter, a continuous pump (Alzet osmotic pump, model 1003D) was placed s.c. in the back area and the infusion cannula (3.6-mm depth) was fixed with the dental cement. The excess part of the implanted cannula was cut with a circular drill head. The osmotic pump was filled with 100 µL **Gd**_**2**_**L**^**1**^ and **Gd**_**2**_**L**^**2**^ ([Gd^3+^] = 10 mM) in artificial cerebrospinal fluid.

#### Preparation of the remote occluding device.

The remote occluding device consisted of 3 main parts: (*i*) support tubing (PE-160; length 108 cm) with a custom-made connector, (*ii*) intraarterial catheter (PE-50; length 1.5 cm), and (*iii*) occluder (diameter 5-0, length 0.31 mm, 5- to 6-mm silicone coating; Doccol) with its extension (PE-90; length 121 cm). The occluder was fixed with superglue (Loctite 454; Henkel) to the MicroTight sleeve (F-183; IDEX Health & Science) that was connected to the extension, and subsequently passed through the support tubing. After the custom-made connector and intraarterial catheter were combined, the occluder was advanced/withdrawn to a desired depth.

#### Preparation of the remote transient middle cerebral artery occlusion.

The rat was repositioned into the MRI bed in supine position. A midline neck incision of ∼2 cm was made and the right common carotid artery (CCA) bifurcation was exposed. The occipital artery was double-ligated (7-0) and dissected. The internal carotid artery (ICA) was isolated rostral until bifurcation with the pterygopalatine artery (PPA). The CCA and the external carotid artery (ECA) were ligated (7-0) and the ICA was temporary clipped. Arteriotomy was carried out on the distal part of the CCA and an intraarterial catheter, filled with heparin, was introduced into the CCA until reaching the ECA–ICA bifurcation, and then fixed with two ligations (4-0). Support tubing was passed through the skin close to the neck incision and additionally fixed with tape along the abdomen of the animal. Thereafter, the intraarterial catheter was connected with support tubing and the occluder was then advanced until the clip positioned on the ICA. After removing the clip, the occluder was quickly advanced until 2 mm after bifurcation with the PPA; subsequently, the ICA was ligated with 7-0 ligature. Lastly, the wound was closed and the animal was transferred inside the scanner where the temperature, breathing rate, heart rate, and blood oxygen saturation were monitored during the scanning. For the occlusion of the MCA, the end of the occluder extension was remotely advanced for 6 to 8 mm, until resistance was felt, indicating that the occluder has reached the anterior carotid artery (ACA) and blocked the blood flow from the ACA and posterior carotid artery (PCA) to the MCA. For reperfusion, the occluder was withdrawn by the same length.

## Supplementary Material

Supplementary File
