# Supplementary material for: Early detection and monitoring of cerebral ischemia using calcium-responsive MRI probes
Source: Proc Natl Acad Sci U S A. 2019 Sep 23;116(41):20666–71. doi: 10.1073/pnas.1908503116 (PMC6789561; doi:10.1073/pnas.1908503116)
Supplement: Supplementary File [file pnas.1908503116.sapp.pdf]

Supporting Information for

Early detection and monitoring of cerebral ischemia using calcium-responsive MRI probes

Tanja Savić,[a] Giuseppe Gambino,[a] Vahid S. Bokharaie,[b] Hamid R. Noori,[b] Nikos K. Logothetis,[c,d,*] and Goran Angelovski[a*]

[a]MR Neuroimaging Agents Group, [b]Neuronal Convergence Group, [c]Department of Physiology of Cognitive Processes, Max Planck Institute for Biological Cybernetics, Tuebingen, Germany

[d]Department of Imaging Science and Biomedical Engineering, University of Manchester, Manchester, United Kingdom

*Correspondence to:

nikos.logothetis@tuebingen.mpg.de

goran.angelovski@tuebingen.mpg.de

Contents

Supplementary Methods S2
Supplementary Figures S7
Supplementary Tables..S14

www.pnas.org/cgi/doi/10.1073/pnas.1908503116

Supplementary Methods

Preparation of Gd_2L^1 and Gd_2L^2. Commercially available reagents and solvents were used without further purification. Purification by column chromatography was performed with silica gel 60 (0.03−0.2 mm) from Carl Roth (Germany). ^1H and ^{13}C NMR spectra were recorded on a Bruker Avance III 300 MHz spectrometer at 25 °C. High resolution mass spectra were recorded on a Bruker Daltonics APEX II (FT-ICR-MS) with an electrospray ionization source. **Gd_2L^1** (Ca-responsive contrast agent) was prepared as previously reported (1). The compound **L^2 (**non-responsive contrast agent) was prepared according to the reaction scheme presented in the Supplementary Fig. 1. Compound **1** was prepared as previously reported(2). Compound **2** (0.31 g, 0.82 mmol) was dissolved in acetonitrile (20 mL) in a 50 mL round bottom flask. Potassium carbonate (0.45 g, 3.28 mmol) was then added as a solid to the stirring mixture, followed by a solution of **1** (1.6 g, 1.97 mmol) in acetonitrile (10 mL). The reaction mixture was stirred for 16 hours at 60 °C. The resulting mixture was cooled, the inorganic salts were removed by filtration and the solution was evaporated *in vacuo*. The crude product was purified by column chromatography (silicagel, 0-15% MeOH in CH_2Cl_2) to obtain pure **3** as a yellow oil (530 mg, 35% yield). ^1H NMR ($CDCl_3$, 300 MHz) δ: 1.46 (s, 72H, CH_3), 2.23 (b, 28H, CH_2), 2.86 (b, 26H, CH_2), 3.37-3.71 (b, 30H, CH_2), 7.12-7.18 (b, 4H, CH_{Ar}), 7.56-7.73 (b, 4H, CH_{Ar}); ^{13}C NMR ($CDCl_3$, 125 MHz) δ: 28.0 (CH_3), 35.2 (CH_2Ph), 40.4 (CH_2NH), 48.7 (CH_2N), 51.8 (CH_2N), 55.6 (CH_2COO), 56.3 (CH_2COO), 70.2 (CH_2O), 81.7 (C_qCH_3), 119.7 (CH_{Ar}), 129.3 (CH_{Ar}), 136.0 (C_{Ar}), 169.6 (COO), 171.9 (COO), 172.9 (COO). HRMS-ESI: calculated for $C_{94}H_{161}N_{14}O_{22}Na_2^{3+}$ $[M+H+2Na]^{3+}$: 628.0563, found: 628.0556.

Synthesis of L^2**.** Compound **3** (35 mg, 0.019 mmol) was dissolved in formic acid (5 mL) in a 25 mL round bottom flask and stirred for 16 hours at 55 °C. The reaction mixture was cooled

and the solvent was evaporated *in vacuo*. The obtained solid was dissolved in water and the pH adjusted to 7.4. After filtration through a 450 nm PTFE filter, the solution was freeze-dried, providing $\mathbf{L^2}$ as a pale yellow solid in quantitative yield. ^{1}H NMR (D_2O, 300 MHz) δ: 2.74-2.79 (b, 10H, CH_2N), 2.91-3.02 (b, 14H, CH_2N), 3.27 (b, 20H, CH_2), 3.36 (b, 10H, CH_2), 3.47 (b, 10H, CH_2), 3.56-3-73 (b, 12H, CH_2), 7.23-7.26 (b, 4H, CH_{Ar}), 7.38-7.41 (b, 4H, CH_{Ar}); ^{13}C NMR ($CDCl_3$, 125 MHz) δ: 34.1 (CH_2Ph), 39.8 (CH_2NH), 47.9 (CH_2N), 48.3 (CH_2N), 51.1 (CH_2N), 51.7 (CH_2N), 54.6 (CH_2N), 55.2 (CH_2N), 56.2 (CH_2COO), 56.8 (CH_2COO), 59.3 (CH_2COO), 68.3 (CH_2O), 69.4 (CH_2O), 121.5 (CH_{Ar}), 129.8 (CH_{Ar}), 135.0 (C_{Ar}), 136.4 (C_{Ar}), 170.3 (COO), 171.2 (COO), 172.7 (COO), 178.0 (COO), 178.9 (COO). HRMS-ESI: calculated for $C_{62}H_{91}CaK_2N_{14}O_{22}^-$ $[M-5H+Ca+2K]^-$: 1501.5338, found: 1501.5330.

Complexation of L^2 with Gd^{3+}. Ligand $\mathbf{L^2}$ (26 mg, 0.019 mmol) was dissolved in 5 mL of water in a 10 mL round bottom flask and the pH was adjusted to 7.4. Aqueous $GdCl_3 \cdot 6H_2O$ (14 mg, 0.038 mmol, dissolved in 1 ml of water) was added portion-wise to the stirring mixture, while maintaining the pH at 7.4 with the addition of aqueous NaOH (1 M). The solution was stirred overnight at room temperature. Subsequently, the solution was treated twice with Chelex® to remove any excess Gd^{3+}. The pH was again adjusted to 7.4 and the solution filtered through 0.2 μm filters and freeze dried, obtaining $\mathbf{Gd_2L^2}$ as a white solid in quantitative yield. HRMS-ESI: calculated for $C_{62}H_{88}Gd_2N_{14}O_{22}^{2-}$ $[M-2H]^{2-}$: 848.2345, found: 848.2373.

The concentration of Gd^{3+} in analysed solutions was determined using the bulk magnetic susceptibility shift method (3).

Relaxometric titrations with Gd_2L^1 and Gd_2L^2. Two solutions of $\mathbf{Gd_2L^1}$ and $\mathbf{Gd_2L^2}$ with starting $[Gd^{3+}]$ = 1.0 mM ([SCA] = 0.5 mM) were titrated using a 5 mM $CaCl_2$ solution, measuring longitudinal relaxation T_1 times after every $CaCl_2$ addition with a 7T (300 MHz)

Bruker Avance III NMR spectrometer at 37 °C. The T_1 times were obtained using standard inversion recovery with a 1% gradient to eliminate the radiation damping effect. The obtained relaxation times were converted into relaxivities (r_1 values) and plotted as a function of Ca^{2+} concentration normalized to an SCA concentration (Equiv. Ca^{2+} = [Ca^{2+}] / [SCA]). The longitudinal relaxivities of the probes were determined according to Equation: $1/T_{1,obs} = 1/T_{1,d} + r_1 \times [Gd^{3+}]$, where $T_{1,obs}$ – the determined longitudinal relaxation time, $T_{1,d}$ – the diamagnetic longitudinal relaxation time (T_1 in the absence of complex) and [Gd^{3+}] – the concentration of Gd^{3+} (equal to double the concentration of the complex) used in the experiment.

Data analysis. Images were scaled and converted to Nifti format. `sinc` interpolation from the `flirt` program in FSL (v 5.0) package was used for motion correction (4). Motion was estimated from interleaved T_1-weighted images and motion correction was conducted using FSL. The images were reconstructed and analyzed using an in-house software written in python v3.6 (5). In each experiment, T_1-weighted signals were recorded. Only experimental sessions which included corrupted signals caused by technical issues were excluded. The sources of these technical issues were either malfunction of the scanner or malfunction of the injection apparatus. Subsequently, each set of T_1-weighted signals was normalized to their maximum value. Cluster analyses were performed to define appropriate regions of interest (ROIs). This allows the identification of a representative signal for consistent investigation of spatiotemporal differences among the contrast agents. Thereby, all the voxels in a large neighborhood (up to the size of the whole hemisphere) of the epicenter of injection were considered at first. Subsequently, K-means clustering, as implemented in scikit-learn library, was applied to the normalized signals (6). K-means algorithm requires the number of clusters as an input. In order to ensure the robustness of the analysis with respect to the choice of parameter K, clustering was conducted for K ranging from 2 to 10 for all datasets and the

results were statistically compared. Thereby, K=2 presented the optimal choice. The regions of interest (ROIs) were then defined using a hierarchical clustering algorithm.

The algorithm scheme is as follows:

1. Apply the K-means clustering algorithm with two clusters to all the voxels in the initial mask;

2. Select all the voxels corresponding to the centroid with a larger mean value;

3. Apply K-means with two clusters to the voxels selected in step 2;

4. Choose the voxels corresponding to the centroid with a larger mean value, and use them as our ROI.

The critical advantage of this algorithm is that it identifies ROIs systematically and without *a priori* assumptions on the number of voxels or total mask size. Here, the algorithm leads to ROIs with approximately 66 voxels in all datasets (Mean: 66.3, SD: 23.9). It is noteworthy that in all datasets, the ROI consist of the voxels in connected neighborhood of the epicenter of injection. The average of all the signals in the identified ROI was used as the representative signal for that dataset.

The representative signals obtained from different experiments have slightly different initial slopes. To remove the effects of small differences in the transient behavior of the representative signals while maintaining the possible qualitative differences in the signals during the ischemic period, a five-step de-trending algorithm was developed (see Fig. S6 for examples of detrending of representative signals):

1. Normalization: The representative signals $s(t)$ are normalized to the respective initial values. The new signal $s^{*}(t)$ represents percentage of changes from the first time-step.

2. Segmentation: The normalized signal ($s^*(t)$) is split along the time-axis into three segments associated with experimental procedures. The first segment $s^*_1(t)$ contains the values corresponding to time-steps from the beginning of the measurement until the onset of ischemia; The second segment $s^*_2(t)$ contains values corresponding to all time-steps during the ischemia; The third segment $s^*_3(t)$ contains all time-steps after the removal of ischemia until the end of measurement.

3. Ignore: Time-series $f(t)$ of identical length as $s^*(t)$ is generated using $s^*_1(t)$ and $s^*_3(t)$, ignoring values from second segment, while keeping the original time-stamps. In other words, $f(t)$ contains data from $s^*(t)$ as if no measurements were done during ischemia while keeping track of time.

4. Interpolation: Cubic splines (i.e. piecewise polynomial functions of degree 3 in variable t) were used to interpolate the non-linear time-series $f(t)$. For this purpose, `splev` function from Scipy library in python(7) was used (smoothing factor of $s=1$) to identify the B-spline representation of all segments of the 1D-curve $f(t)$ including segment 2 ($f_{sp}(t)$). Thereby, the spline function $f_{sp}(t)$ is extrapolated over segment 2 assuming as if it follows the same trend as is inherent in segments 1 and 3.

5. Detrending: The difference $d_s(t)=f_{sp}(t)-s^*(t)$ provides the detrended signal.

For non-ischemic experiments, the detrending procedure is identical to the above algorithm, with the difference that step 3 (ignore) is not performed. The spline function with segment 2 is then based on data from the experiment and not, as in case of ischemia, based on the obtained trends from segments 1 and 3.

Supplementary Figures

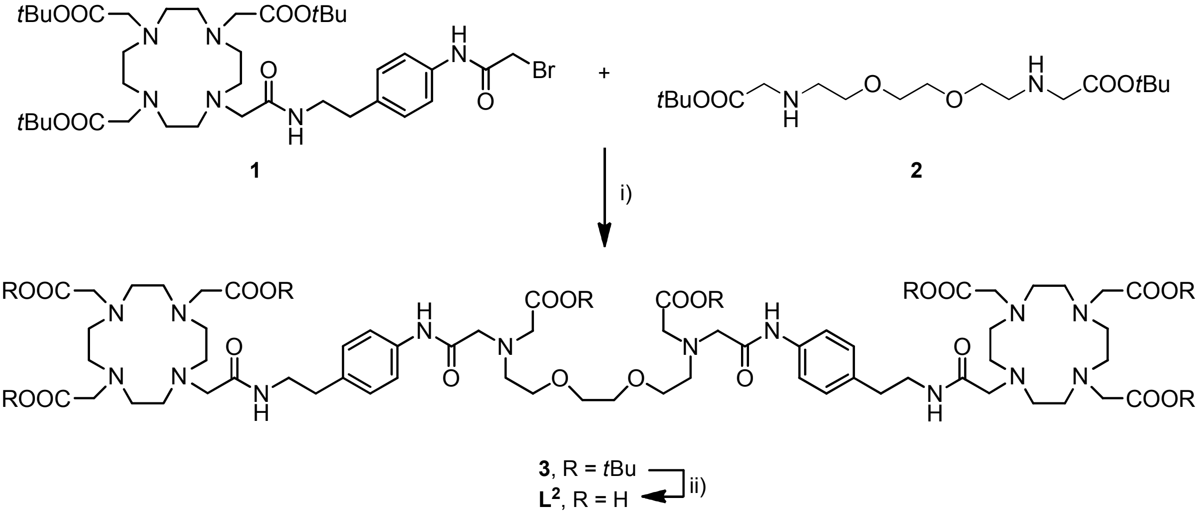

Supplementary Figure 1: Reaction scheme for the synthesis of $\mathbf{L^2}$; i) K_2CO_3, MeCN, 60 °C, 16 h; ii) HCOOH, 55 °C, 16 h.

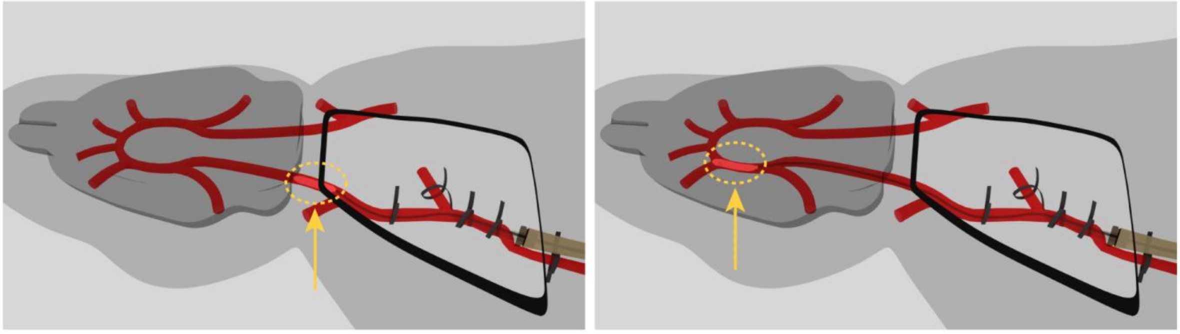

Supplementary Figure S2. Positioning of the occluder in pre-and post-ischemia period (left) and during the MCAo (right).

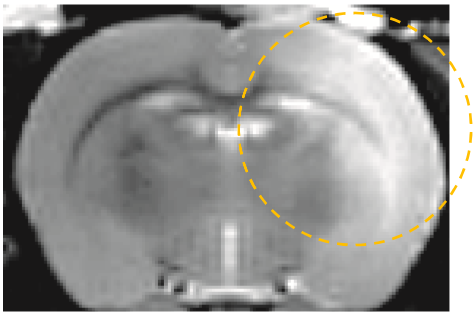

Supplementary Figure 3. Confirmation of cerebral ischemia, T_2-weighted image acquired after tMCAo performed on right MCA. Orange dashed circle marks the area affected by stroke.

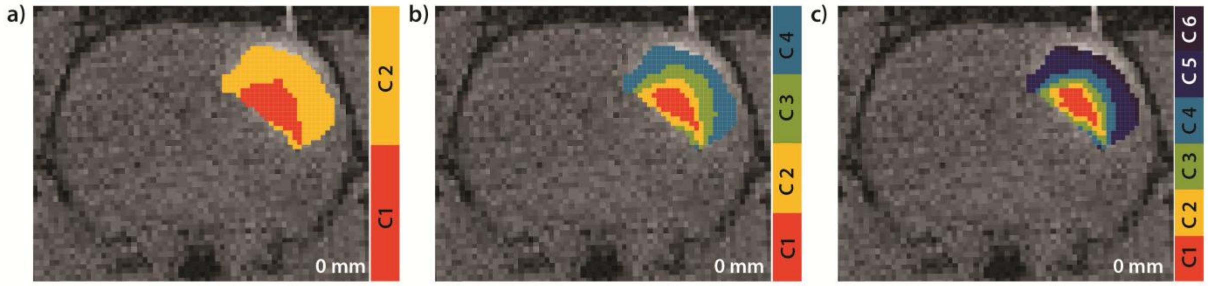

Supplementary Figure 4. Comparison of K-means clustering of the responsive $\mathbf{Gd_2L^1}$ infused in the somatosensory cortex. T_1-weighted image overlayed with cluster maps done using cluster numbers of a) K=2, b) K=4, and c) K=6.

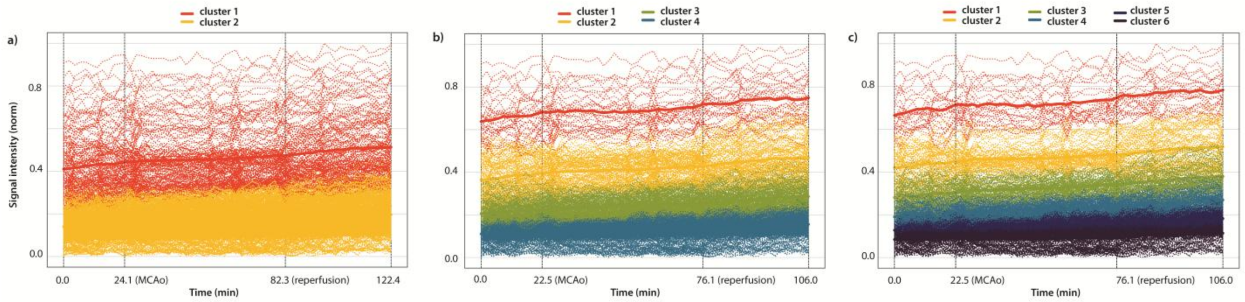

Supplementary Figure 5. Centroids and the corresponding signals for cluster maps shown in Figure S3.

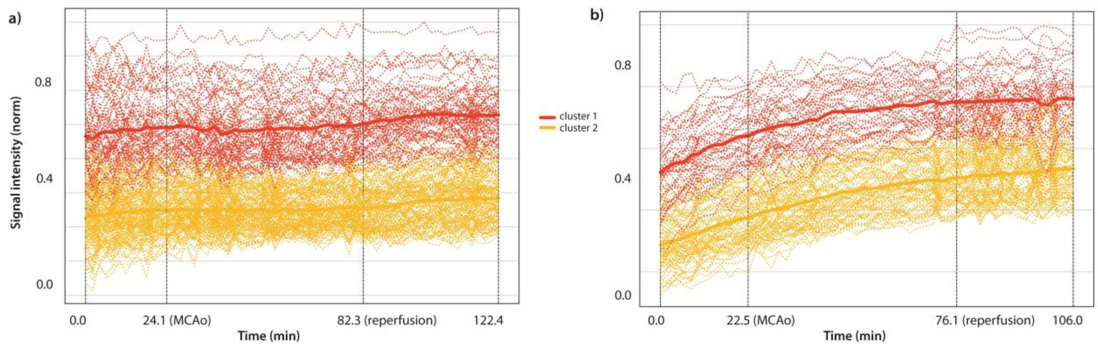

Supplementary Figure 6. Centroids and the corresponding signals for cluster maps shown in Figure 3c.

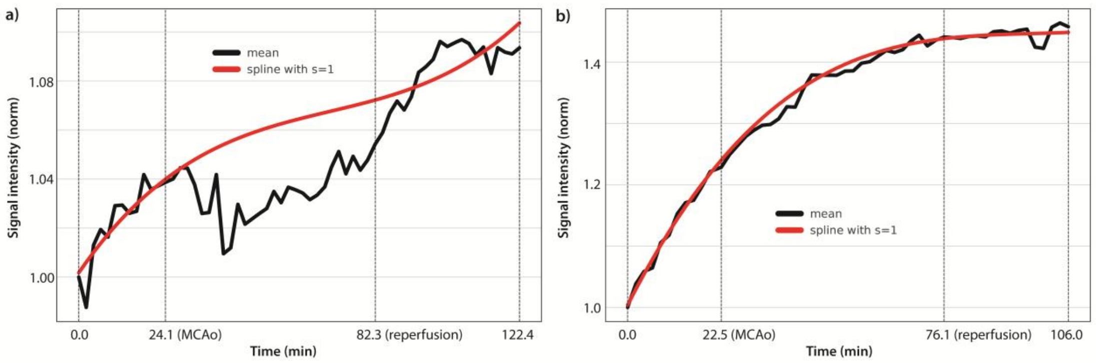

Supplementary Figure 7. Examples of de-trending of representative signals: a) **Gd_2L^1** b) **Gd_2L^2**.

Supplementary Tables

Supplementary Table 1. Gd_2L^1 mean values of de-trended signals for experiments with cerebral ischemia.

**Detrended signal mean Gd_2L^1**	**Pre-ischemia** (10^{-4})	**Ischemia** (10^{-2})	**Post-ischemia** (10^{-4})
exp 1	4.15	-1.29	-2.18
exp 2	6.17	-2.44	-3.39
exp 3	4.26	-3.29	-3.13
exp 4	4.43	-4.41	-3.55
exp 5	1.98	-1.79	-1.58

Supplementary Table 2. Gd_2L^2 mean values of de-trended signals for experiments with cerebral ischemia.

**Detrended signal mean Gd_2L^2**	**Pre-ischemia** (10^{-5})	**Ischemia** (10^{-2})	**Post-ischemia** (10^{-5})
exp 1	2.06	-0.10	-1.51
exp 2	-4.21	1.68	3.09
exp 3	-5.39	-0.75	3.95
exp 4	-6.26	-0.23	5.01
exp 5	-5.00	0.34	3.67

1. Angelovski G*, et al.* (2008) Smart magnetic resonance imaging agents that sense extracellular calcium fluctuations. *ChemBioChem* 9(11):1729-1734.

2. Connah L & Angelovski G (2018) Synergy of Key Properties Promotes Dendrimer Conjugates as Prospective Ratiometric Bioresponsive Magnetic Resonance Imaging Probes. *Biomacromolecules* 19(12):4668-4676.

3. Corsi DM, Platas-Iglesias C, van Bekkum H, & Peters JA (2001) Determination of paramagnetic lanthanide(III) concentrations from bulk magnetic susceptibility shifts in NMR spectra. *Magn. Reson. Chem.* 39(11):723-726.

4. Jenkinson M, Beckmann CF, Behrens TE, Woolrich MW, & Smith SM (2012) FSL. *Neuroimage* 62(2):782-790.

5. Bokharaie VS (2019) MiTfAT: A Python-based fMRI Analysis Tool. Zenodo. https://doi.org/10.5281/zenodo.3372365.

6. Pedregosa F*, et al.* (2011) Scikit-learn: Machine Learning in Python. *J. Mach. Learn. Res.* 12:2825-2830.

7. Jones E, Oliphant T, & Peterson P (2001) SciPy: Open source scientific tools for Python. in *http://www.scipy.org/*.
